# Impact of biosolids amendment and wastewater effluent irrigation on enteric antibiotic-resistant bacteria – a greenhouse study

**DOI:** 10.1016/j.wroa.2021.100119

**Published:** 2021-09-08

**Authors:** Catherine Mays, Gabriela L. Garza, Joy Waite-Cusic, Tyler S. Radniecki, Tala Navab-Daneshmand

**Affiliations:** a School of Chemical, Biological, and Environmental Engineering, Oregon State University, 105 SW 26th St, 116 Johnson Hall, Corvallis, OR 97331, United States; b Department of Food Science and Technology, Oregon State University, 3051 SW Campus Way, Corvallis, OR 97331, United States

**Keywords:** Antibiotic resistance, Biosolids amendment, Wastewater irrigation, *Escherichia coli*, Enterococci

## Abstract

Reuse of wastewater effluent and biosolids in agriculture is essential to sustainable water and nutrient resource management practices. Wastewater and biosolids, however, are reportedly the recipients, reservoirs, and sources of antibiotic-resistant enteric pathogens. While decay rates of fecal bacterial indicators in soil are frequently studied, very few studies have reported on the persistence of the antibiotic-resistant sub-populations. Little is known about how multi-drug resistance phenotypes of enteric bacteria in agricultural soil change over time. In this study, germinated carrot seeds were planted in soil that received biosolids amendment and/or wastewater effluent irrigation in a greenhouse setting. We quantified total and antibiotic-resistant fecal bacterial indicators (*Escherichia coli* and enterococci) weekly in soil and total *E. coli* at harvest (day 77) on carrots. Antibiotic susceptibility of 121 *E. coli* and 110 enterococci collected isolates were determined. *E. coli* or enterococci were not recovered from the soil without biosolids amendment regardless of the irrigation water source. After biosolids amendment, soil *E. coli* and enterococci concentrations increased more than 3 log_10_ CFU/g-TS within the first week, declined slowly over time, but stayed above the detection limit (0.39 CFU/g-TS) over the entirety of the study. No statistical difference was found between effluent wastewater or water irrigation in soil total and antibiotic-resistant *E. coli* and enterococci concentrations or carrots *E. coli* levels. Soil antibiotic-resistant *E. coli* and enterococci decayed significantly faster than total *E. coli* and enterococci. Moreover, the prevalence of multi-drug resistant (resistance to three or more antibiotics) *E. coli* declined significantly over time, while almost all collected enterococci isolates showed multi-drug resistance phenotypes. At harvest, *E. coli* were present on carrots; the majority of which were resistant to ampicillin. The survival of antibiotic-resistant enteric bacteria in soil and on harvested carrots indicates there are transmission risks associated with biosolids amendment use in root crops.

## Highlights

• Wastewater irrigation did not impact soil antibiotic resistance bacterial levels.
• Biosolids soil amendment increased antibiotic-resistant *E. coli* and enterococci.
• Antibiotic-resistant bacteria persisted in soil with presence on carrots at harvest.
• Antibiotic-resistant bacteria decayed faster than total counterparts.

## Graphical abstract

**Figure:**
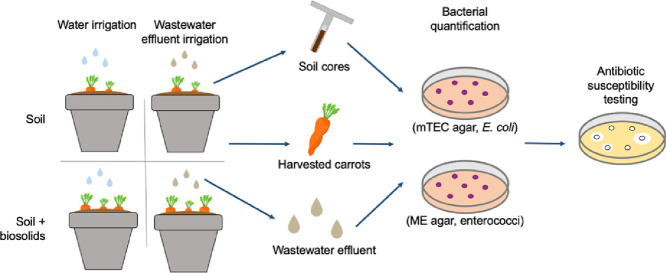

## 1 Introduction

The world's population is expected to reach nearly 10 billion by the year 2050, requiring an increase in agricultural production of over 60% from 2005 (United Nations, 2019). To keep up with this rise in global food demand, the use of treated wastewater effluent (TWE) for irrigation as an alternative water resource, and animal or human excreta (i.e., manure or biosolids) as soil amendments is increasing. One of the main human health risks associated with the application of wastewater effluent and biosolids in agriculture is the potential contamination of food crops with infectious enteric bacteria. In addition to their infectivity, the emergence of enteric bacteria resistant to one or more antibiotics in municipal wastewater and biosolids and, consequently, their receiving environments is a growing human health concern (E.U. Commission, 2017). Each year in the U.S. alone, there are 2.8 million infections with antibiotic-resistant bacteria (ARB) with 35,000 deaths as a direct result of these infections (CDC, 2019). The human health impacts of the presence and dissemination of enteric ARB in the environment are not well-defined. Consequently, the microbial regulations which exist for agricultural use of TWE and biosolids to protect human welfare may be insufficient to mitigate the risk associated with antibiotic resistance in agricultural systems. Identifying the exposure risks of antibiotic resistance due to wastewater and biosolids application in agriculture is essential to food safety and human health (Ashbolt et al., 2013).

Significant levels of ARB are present in both biosolids (∼5–11 log_10_ CFU/g) and TWE (∼0.7–4 log_10_ CFU/mL), even after the use of advanced treatment technologies, such as membrane bioreactors or anaerobic digesters (Guardabassi et al., 2002; Munir et al., 2011; Novo et al., 2013; Smyth et al., 2020). The subsequent use of these ARB-laden resources in agricultural settings has raised concerns about their safety (Christou et al., 2017; Michael et al., 2013). Some studies on the prevalence of ARB on food crops grown in amended soil or irrigated with TWE have not detected their presence (Rahube et al., 2014), while others report ARB and their determinant genes on food crops (Marti et al., 2013; Micallef et al., 2013). Yet, there is limited knowledge on the fate (prevalence, growth, and persistence) of enteric ARB in soil and on food crops. Moreover, the potential changes in phenotypic antibiotic susceptibility profile of these resistance bacteria after biosolids amendment and/or continued TWE irrigation is not clearly understood. The risk of exposure to enteric ARB and multi-drug resistance (i.e., resistant to three or more classes of antibiotics, MDR) bacteria as food moves from the farm to the table and the subsequent clinical impact on increased illness, disability, and death demonstrate the need to determine the survival of enteric ARB and their non-ARB counterparts in soil environments.

In this work, we aim to determine the fate of antibiotic-resistant fecal indicator bacteria, and the change in their phenotypic characterization over time in agricultural soil after biosolids amendment and/or TWE irrigation. To achieve these objectives, different combinations of biosolids amendment and TWE irrigation were applied to soil in a controlled greenhouse setting during carrot production (i.e., eleven weeks). Cultivatable total and antibiotic-resistant *E. coli* and enterococci (Gram-negative and Gram-positive fecal bacterial indicators) were quantified in biosolids and TWE and tracked weekly in soil. Declining populations of ARB versus susceptible counterparts were determined, and the presence of *E. coli* on carrot skins at harvest was measured. Finally, the phenotypes of antibiotic susceptibility of presumptive *E. coli* and enterococci colonies and the prevalence of MDR enteric bacterial indicators over the growing period were identified.

## 2 Methods

### 2.1 Greenhouse study design

This study was conducted in a greenhouse at Oregon State University (Corvallis, OR) between February and May 2019. The greenhouse consisted of 1.5 *m* × 4.5 m steel mesh grid tables on which 1.2 *m* × 1.2 m growth trays were placed to collect and contain any runoff. Polyvinyl chloride (PVC) pipe cages with mosquito netting were constructed around each tray to prevent flying insects from accessing the plants and soil. In the greenhouse, the ambient temperature was maintained at approximately 15 °C overnight and fluctuated between 20 °C and 26 °C during the daylight hours. Soil was obtained from a commercial agricultural field from the Willamette Valley area in Oregon with no history of wastewater irrigation or biosolids/manure amendment. The collected soil was air-dried and passed through a 5-mm sieve.

The study design included four treatment groups, consisting of soil with or without biosolids amendment that were irrigated with TWE or deionized (DI) water. There were six replicate pots in each treatment group that were randomly assigned to two groups (triplicates). On each sampling day, we rotated between the two groups for testing. Plastic nursery containers (7.5 L) were filled with approximately 5.8 kg of soil. Pots in the biosolids amendment treatment groups were amended with dewatered biosolids at a ratio of 70 g/kg of soil (1% dry w/w) the day prior to planting. Carrot seeds were obtained from a local farm and were germinated by keeping them in moist paper towels at room temperature for 12 d.

Four germinated seeds were sown into each pot. Pots were irrigated with TWE or DI water 2 to 3 times per week. Irrigation water was applied to maintain a soil moisture content of 80–85% total solids (150–500 mL adjusted for measured solids content) and to minimize the draining of water from the bottom of the pots.

### 2.2 Collection and storage of biosolids, effluent, soil, and carrots

Municipal biosolids and TWE were collected from a local water reclamation facility in Oregon. This facility receives municipal wastewater from a serving population of approximately 55,000 with average daily flow rates of 8–10 million gal. Treatment methods include a traditional activated sludge basin, secondary clarifiers, and chlorination by sodium hypochlorite. Settled sludge enters a holding tank prior to dewatering via belt press. Dewatered Class B biosolids were collected in the beginning of the study, transferred to the laboratory on ice. Upon arrival to the laboratory, the collected biosolids were processed for pH, total solids, and bacterial enumerations. Biosolids were stored at 4 °C overnight, brought to the greenhouse, kept at room temperature, and applied to appropriate study pots within 9 h. TWE was collected weekly from the water reclamation facility prior to discharge, transferred to the laboratory on ice and stored at 4 °C for up to 7 d before use.

Each week, soil core samples were collected in triplicate before irrigation. Soil cores were collected from bulk soil (defined as soil not penetrated by plant roots) from the soil surface to an approximate depth of 25 cm using a soil sampler probe (M.K. Rittenhouse & Sons Ltd., St. Catharines, Ontario, Canada) to account for the possibility of vertical bacterial migration. On day 0, soil samples were collected after all pots were filled with appropriate soil and biosolids amendment. All soil samples were collected prior to irrigation. Collected soil was placed into a sterile WhirlPak bag (Nasco, Fort Atkinson, WI) and homogenized by massaging and shaking for 2 min. Soil bags were stored on ice and processed for pH, total solids, and bacterial enumeration within 24 h.

After 11 weeks (77 d), carrots were harvested from pots. To harvest, the entirety of the pot contents was gently removed and placed into a containment bin. Bulk soil was brushed aside until carrots could be removed. Carrot tops were removed using sterile scissors and the carrots from each pot were then placed into a bag. The carrot roots were stored at 4 °C and processed for bacterial enumeration within 24 h.

### 2.3 Physical and chemical parameters

Soil total solids were measured on each sampling day following the Standard Methods for the Examination of Water and Wastewater 2540B (APHA et al., 2017). Soil pH was measured biweekly by mixing bulk soil and DI water at a ratio of 1:9. The mixture was stirred for 5 min before measurement using a pH probe (VWR, Radnor, PA).

### 2.4 Bacterial enumeration and isolation

Initial biosolids and weekly soil samples were mixed 1:1 with phosphate buffered saline (PBS), shaken for 2 min, and allowed to settle for an additional 2 min prior to preparing serial dilutions in PBS. Carrot samples were rinsed with 40 mL PBS by shaking and were subsequently massaged for 60 s. Soil supernatant and carrot rinsate were spread plated onto prepared agar plates. Additionally, aliquots of soil dilutions (1 mL), carrot rinsate (10 mL), and TWE (200 mL) were filtered through 0.45 µm mixed-cellulose ester membranes (Whatman, Kent, UK) and the filters were placed on prepared agar plates. To enumerate presumptive *E. coli*, mTEC ChromoSelect (Sigma Aldrich, St. Louis, MO) was used. Antibiotic-resistant presumptive *E. coli* were quantified using mTEC agar supplemented with the intermediate breakpoint concentrations of ampicillin (16 µg/mL), ciprofloxacin (2 µg/mL), or tetracycline (8 µg/mL; Hardy Diagnostics, Santa Clara, CA) (CLSI, 2018). After spread plating or membrane filtration, mTEC plates were inverted and incubated for 22 to 24 h at 44.5 °C. Purple or magenta colonies were counted as total or antibiotic-resistant presumptive *E. coli*. Presumptive enterococci were quantified using m-Enterococcus agar (Hardy Diagnostics, Santa Maria, CA). To enumerate antibiotic-resistant enterococci, m-Enterococcus agar were supplemented with ampicillin (8 µg/mL) or tetracycline (8 µg/mL) (CLSI, 2018). m-Enterococcus agar plates were inverted and incubated for 48 h at 36 °C prior to enumeration. Dark red or maroon colonies were counted as presumptive enterococci. In biosolids and soil, bacterial concentrations were calculated as log_10_ colony forming units per g total solids (log_10_ CFU/g-TS) with a lower detection limit of 0.39 log_10_ CFU/g-TS. For TWE, bacterial levels are reported as log_10_ CFU/mL and the lower limit of detection was −2.3 log_10_ CFU/mL. Carrot bacterial concentrations were calculated as log_10_ CFU/40 mL-rinsate with a lower detection limit of 0 log_10_ CFU/10 mL-rinsate.

Presumptive *E. coli* and enterococci colonies were randomly selected from antibiotic-supplemented plates during days 0 and 35 (no colonies were detected on supplemented plates on day 77). Day 0 isolates were collected after biosolids amendment and prior to irrigation. At harvest, presumptive *E. coli* colonies from both soil and carrots were selected from non-supplemented plates. Collected *E. coli* and enterococci isolates were grown overnight at 37 °C in 1 mL LB and TSB broth, respectively, supplemented with 20% glycerol, and stored at −20 °C until further processing.

### 2.5 Antibiotic susceptibility testing of isolates

Antibiotic susceptibility of the collected *E. coli* and enterococci isolates was determined using the disk diffusion method (Bauer et al., 1966). Briefly, isolates were grown in Mueller-Hinton broth (Hardy Diagnostics, Santa Maria, CA) until the concentration visually matched a 0.5 MacFarland standard (Hardy Diagnostics, Santa Maria, CA). Suspensions were spread onto Mueller-Hinton agar using a sterile swab, and disks containing the target antibiotics (Hardy Diagnostics, Santa Clara, CA) were placed onto each plate. Target antibiotics were chosen to include multiple antibiotic classes and mechanisms of action. *E. coli* isolates were tested against ampicillin (10 µg), chloramphenicol (30 µg), ciprofloxacin (5 µg), gentamycin (10 µg), tetracycline (30 µg), trimethoprim-sulfamethoxazole (1.25/23.75 µg); and enterococci isolates were tested against ampicillin (10 µg), ciprofloxacin (5 µg), erythromycin (15 µg), tetracycline (30 µg), and vancomycin (30 µg) (CLSI, 2018). Plates were inverted and incubated for 16 to 18 h at 37 °C. The zone of inhibition around the disks was measured to classify the isolates as susceptible or resistant to the tested antibiotics; isolates classified as intermediate are also reported as “resistant” in this study. Quality control checks were performed between every 25 tests with *E. coli* ATCC 25922 for presumptive *E. coli* isolates and *Staphylococcus aureus* ATCC 25923 for presumptive enterococci isolates (CLSI, 2018).

### 2.6 Data analysis

Mean cell density and standard error of total and antibiotic-resistant *E. coli* and enterococci were calculated in Excel 2016. Other statistical analyses were performed in R (R Statistical Software, version 1.1.456, R Foundation for Statistical Computing, Vienna, Austria). The limit of detection was used for bacterial concentrations at or below the detection limits. Normality of raw data was determined using Shapiro tests. One-way ANOVA or Kruskal-Wallis tests were then used accordingly to determine statistical differences between pH and total solids of treatment groups, as well as differences between irrigation water and bacterial abundance. ANOVA was followed by a post-hoc Tukey's HSD test as appropriate. Comparisons of prevalence of MDR colonies and their correlation with prevalence of different resistance phenotypes (i.e., resistance to individual antibiotics) were achieved using a Pearson's chi-squared test for *E. coli* and Fisher's Exact test for enterococci, the choice of test was based on the sample size for the target bacteria. Statistical difference was defined at α = 0.05.

## 3 Results and discussion

### 3.1 Physical-chemical parameters

**Fig. 1:**
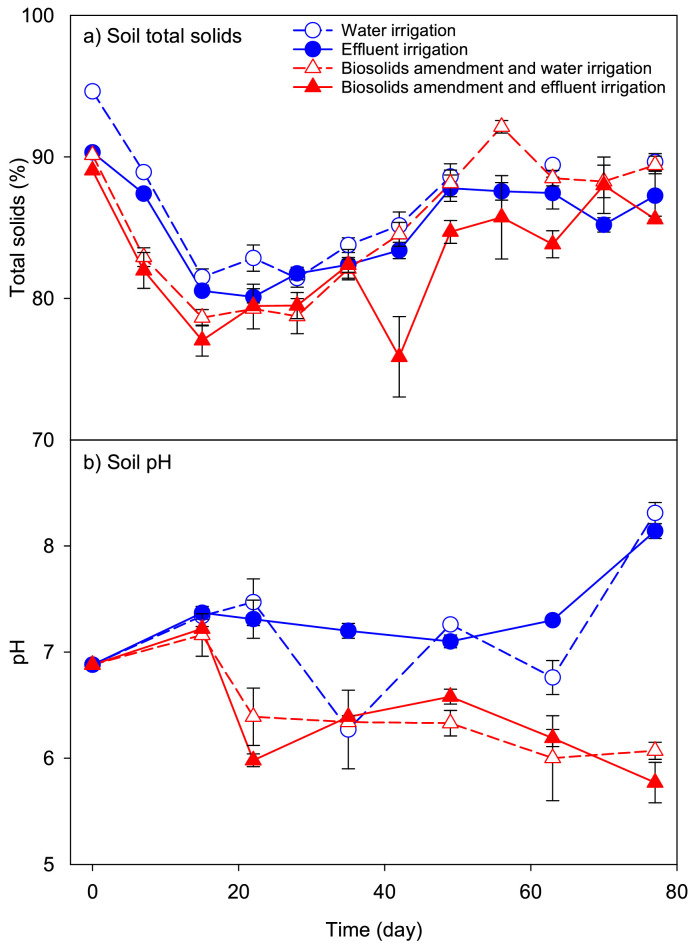
a) Total solids, and b) pH in soil with and without biosolids amendment irrigated with treated wastewater effluent or DI water over 77-day growth period of carrots from seed in a greenhouse setting. Total solids were measured weekly and pH was measured biweekly. On day 0, total solids and pH were measured before irrigation and after biosolids amendment. On all other sampling days, total solids and pH were measured prior to irrigation. Error bars represent standard error of three replicates.

The soil was characterized as loamy sand (83% sand, 13% silt, and 3% clay) with 0.21% carbon, 0.02% nitrogen, pH of 6.88, and an electrical conductivity of 0.057 dS/m (Central Analytical Laboratory, Oregon State University). Total solids in biosolids were 14.4% and in soil ranged from 76.0 to 94.6% in all four treatment groups (i.e., with or without biosolids amendment, and irrigated with DI water or TWE) throughout the study (Fig. 1a). There were significant variations in total solids throughout the study (One-way ANOVA, *p* < 0.01). To maintain a moisture content of 80–85% in soil, irrigation plans were modified weekly after solids analyses. Soil pH was measured biweekly (Fig. 1b). pH was significantly lower in biosolids amended soil (6.35 ± 0.08) compared to non-amended pots (7.29 ± 0.10; Kruskal-Wallis, *p* < 0.001). Irrigation with TWE, however, did not significantly impact soil pH when compared to DI water irrigation (Kruskal-Wallis, *p* > 0.05).

### 3.2 Total and antibiotic-resistant bacteria in biosolids and wastewater

**Fig. 2:**
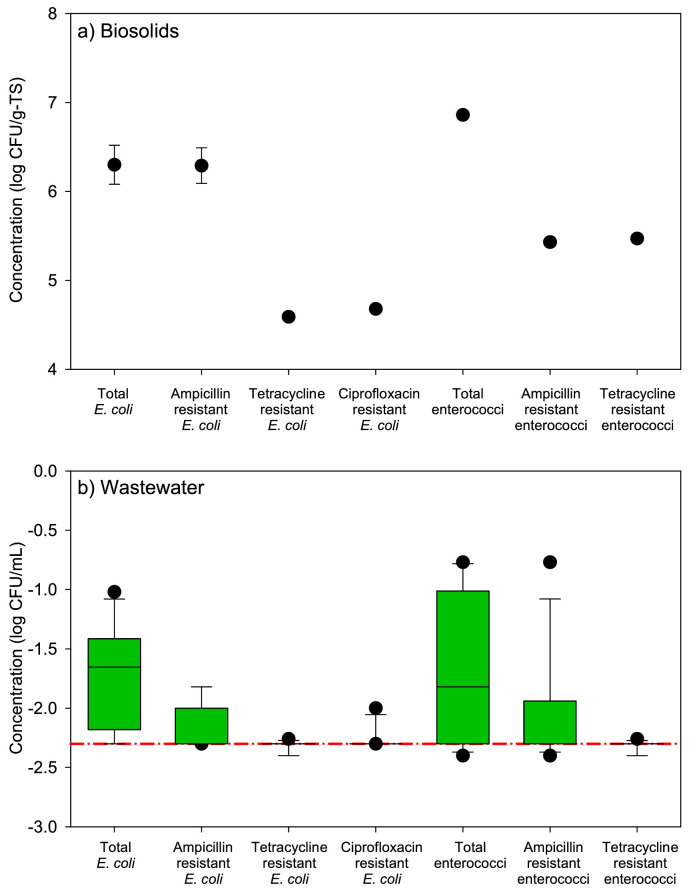
Concentrations of total and antibiotic-resistant presumptive *E. coli* and enterococci in a) biosolids and b) treated wastewater effluent. Biosolids were applied as soil amendment to some treatment groups on day 0 of the greenhouse study. For biosolids data, the error bars represent standard error of three replicates. Treated wastewater effluent was used for irrigation in some treatment groups. The box plots represent the median, 10th, 25th, 75th, and 90th percentiles for eleven treated wastewater effluent samples collected weekly. The lower limits of detection were 0.39 log_10_ CFU/g-TS in biosolids and −2.3 log_10_ CFU/mL in treated wastewater effluent (shown with a red dashed line).

Biosolids contained 6.3 ± 0.2 log_10_ CFU/g-TS of presumptive *E. coli* (Fig. 2a), the majority of which were resistant to ampicillin (6.2 ± 0.2 log_10_ CFU/g-TS). In addition, there were over 4.0 log_10_ CFU/g-TS of tetracycline- and ciprofloxacin-resistant *E. coli* present in biosolids. Biosolids also harbored 6.9 ± 0.0 log_10_ CFU/g-TS enterococci with about 5.5 log_10_ CFU/g-TS resistant to tetracycline and ampicillin.

Concentrations of total *E. coli* and enterococci in TWE irrigation water over the eleven weeks of the study were consistently below −1.0 and −0.8 log_10_ CFU/mL, respectively (Fig. 2b). Culturable antibiotic-resistant *E. coli* and enterococci were detected (limit of detection: −2.3 log_10_ CFU/mL) in nine and seven of the 12 TWE samples during the eleven-week period of the study, respectively (Fig. 2b). The U.S. Food Safety Modernization Act's (FSMA) Produce Safety Rule (PSR) mandates minimum standards for agricultural practices for produce destined for human consumption, including specific sections to mitigate risks associated with agricultural water (Subpart E) and biological soil amendments of animal origin (BSAAO; Subpart F) (Food and Drug Administration, 2015). Under the PSR, water used for pre-harvest activities, including irrigation, must have low levels of generic *E. coli* (geometric mean ≤ 126 colony forming units (CFU)/100 mL water; statistical threshold value < 410 CFU/100 ml). Accordingly, total *E. coli* levels in the TWE were very low (∼2 CFU/100 ml) throughout the study indicating that the TWE from the treatment facility would be of acceptable microbiological quality for use in preharvest application on produce subject to the PSR (Food and Drug Administration, 2015).

While these limits have been codified, the microbial water quality requirements are currently under reconsideration by the FDA (Food and Drug Administration, 2019, 2016).

### 3.3 Survival of total and antibiotic-resistant bacteria in irrigated biosolids-amended soil

**Fig. 3:**
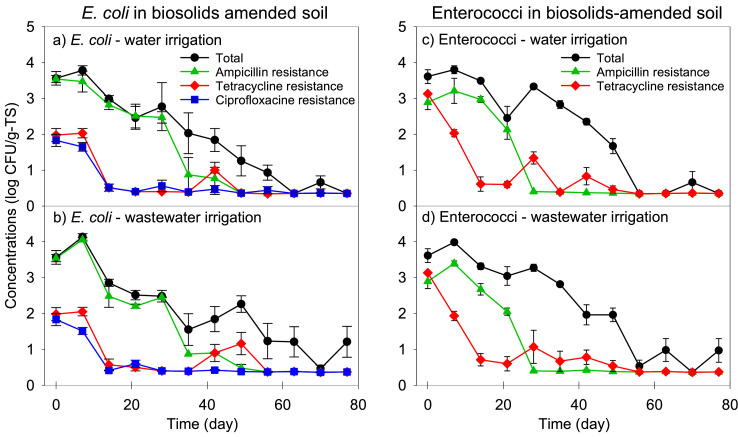
Concentrations of total and antibiotic-resistant presumptive *E. coli* and enterococci in biosolids-amended soil with water or treated wastewater effluent irrigation. Soil samples were collected as cores from pots over 77 days of carrot growth from seeds in a greenhouse. The lower limit of detection was 0.39 log_10_ CFU/g-TS in soil. Error bars represent standard error of three replicates. Results show that irrigation with TWE or DI water did not have an impact on total or antibiotic-resistant *E. coli* or enterococci concentrations in biosolids-amended soil during the course of the study (Kruskal-Wallis, *p* > 0.05; Fig. 3). The slight increase in concentrations of *E. coli* or enterococci a few days after amendment, followed by a period of decay, has been reported in other studies (Montealegre et al., 2018; Sharma and Millner, 2016), and could be due to the initial ability of the bacteria to access and utilize the nutrients and moisture in the soil (Sharma and Reynnells, 2018).

The abundance of culturable total and antibiotic-resistant *E. coli* and enterococci was quantified in irrigated biosolid-amended soil over a 77-day period (Fig. 3). Biosolids amendment increased both *E. coli* and enterococci levels in soil to 3.6 ± 0.2 log_10_ CFU/g-TS. These *E. coli* and enterococci levels in soil (i.e., after biosolids amendment) were about 1 log_10_ CFU/g-TS lower than predicted which is potentially due to the 24-hour period between sample collection and processing on day 0. An increase of 0.58 log_10_ CFU/g-TS was observed in TWE-irrigated pots (Fig. 3b) and 0.23 log_10_ CFU/g-TS in DI-irrigated pots (Fig. 3a) at day 7 after which total *E. coli* levels decreased slowly but remained above the detection limit (0.39 log_10_ CFU/g-TS) over the entirety of the study. Results demonstrated that a large portion of *E. coli* detected in biosolids-amended soil were resistant to ampicillin and that they survived in soil for the first four weeks of the study but declined to below the limit of detection by week eight. Resistance of *E. coli* to tetracycline and ciprofloxacin were present at around 2 log_10_ CFU/g-TS in amended soil but declined quickly and were below the detection limit by day 21.

Enterococci levels slightly increased to 3.98 log_10_ CFU/g-TS in TWE-irrigated soil by day 7, followed by a slow decline over time (Fig. 3d). Enterococci concentrations fluctuated between the detection limit and 1.0 log_10_ CFU/g-TS from day 63 until the end of the study. Compared to *E. coli*, enterococci were more adept at surviving in the biosolids-amended, TWE-irrigated soil environment. Ampicillin-resistant enterococci followed a similar pattern, increasing from an initial concentration of 2.89 log_10_ CFU/g-TS in the biosolids-amended soil to 3.21 log_10_ CFU/g-TS and 3.39 log_10_ CFU/g-TS in DI- and TWE-irrigated soil on day 7, respectively, followed by a decline over the next 3 weeks. Ampicillin-resistant enterococci declined to the detection limit by day 28 and tetracycline-resistant enterococci declined to similar levels by day 56.

Background soil was verified to have no detectable total or antibiotic-resistant *E. coli* or enterococci (< 0.39 log_10_ CFU/g-TS). Samples without biosolids amendment that were irrigated with DI water (controls) had no detectable *E. coli* nor enterococci throughout the course of the study. In the pots without biosolids amendment that received TWE irrigation, total and ampicillin-resistant *E. coli* were detected at 0.74 log_10_ CFU/g-TS on day 14, and the levels were below the detection limit (0.39 log_10_ CFU/g-TS) for the remainder of the study. Hence, no significant difference was observed on the prevalence of total or antibiotic-resistant *E. coli* and enterococci in unamended soil irrigated with DI water and TWE (Kruskal-Wallis, *p* > 0.05).

No statistical difference was observed between the concentrations of *E. coli* and enterococci throughout the study period (Kruskal-Wallis, *p* > 0.05; Fig. 3), suggesting these two fecal indicators behave similarly in soil. The antibiotic-resistant subpopulations decayed at faster rates than their total counterparts for both *E. coli* and enterococci. Tetracycline-resistant *E. coli* decayed at a faster rate compared to total and ampicillin- and ciprofloxacin-resistant *E. coli*. Similarly, tetracycline- and ampicillin-resistant enterococci declined faster than total enterococci populations. Overall, while total and antibiotic-resistant *E. coli* and enterococci levels declined rapidly in soil, our findings demonstrate that antibiotic-resistant *E. coli* and enterococci declined at a faster rate than the total counterparts. To the authors’ knowledge, this study is the first to directly compare declining populations of total and antibiotic-resistant *E. coli* and enterococci in soil receiving biosolids amendment and/or TWE irrigation. Our findings are in line with a study tracking *E. coli* in dewatered, anaerobically digested sludge-amended soil (Lang and Smith, 2007). Other studies of biosolids- and animal manure-amended soils have reported a comparable microbial decline (Lang et al., 2007; Schwarz et al., 2014). The complex nature of the interactions between the soil environment and parameters such as moisture content, temperature, pH, availability of resources (e.g., oxygen, carbon), physical characteristics of soil (e.g., proportions of silt and clay), soil microbiota that affect growth and survival of total and antibiotic-resistant enteric bacteria can account for these differences (Montealegre et al., 2018; Navab-Daneshmand et al., 2014; van Elsas et al., 2011). Moreover, while continuous decline of the soil total and antibiotic-resistant *E. coli* and enterococci were observed, the transfer of resistance to other bacterial species via mechanisms such as horizontal gene transfer should also be considered (Rahube et al., 2016; Riber et al., 2014). Future research could include investigations into whether ARB decay at faster rates due to decreased environmental fitness or due to disposal of the resistance genes on mobile genetic elements into the surrounding environment.

### 3.4 Survival of total *E. coli* on carrots grown in irrigated biosolids-amended soil

*E. coli* concentrations were measured on carrots at harvest. These levels were below the limit of detection (0 log_10_ CFU/10 mL-rinsate) on carrots grown in soil without biosolids amendment. In carrots harvested from biosolids-amended treatment groups, *E. coli* concentrations were 1.0 ± 0.2 log_10_ CFU/40 mL-rinsate under DI water irrigation and 0.8 ± 0.2 log_10_ CFU/40 mL-rinsate under TWE irrigation. Similar to soil data, no statistical difference was found between the two irrigation patterns (one-way ANOVA, *p* > 0.05). Other studies tracking bacterial pathogen indicators in biosolids amended soil have found varying results on their presence on vegetable crops at harvest. Some studies have shown that *E. coli* can survive in soil for lengthy time periods and can be present on the food crops at harvest (Islam et al., 2004). Others have not found significant evidence that biosolids amendment increases the abundance of *E. coli* or enterococci on vegetables at harvest (Rahube et al., 2014). The time between the amendment and harvest appears to be a strong determinate of the survival of pathogenic bacterial indicators on crops, as has been previously reported in literature and is regulated by governing bodies (Food and Drug Administration, 2015; Gale, 2005; U.S. EPA, 1999). The U.S. Environmental Protection Agency categorizes biosolids application to agriculture in part based on their microbiological quality (U.S. EPA, 2003, 1999). Class A biosolids must be treated to a fecal coliform density of < 1000 most probable number per gram of total solids (MPN/g-TS) which may be applied to land with no restrictions (U.S. EPA, 1999). Class B biosolids are allowed higher microbial concentrations (fecal coliform density of < 2 × 10^6^ MPN/g-TS), but restrictions exist for land application, including extended application-to-harvest timeframes (30 days to up to 20 months) (U.S. EPA, 1999). Under the right environmental conditions, fecal indicators, such as *E. coli* and enterococci, may persist for several months in the soil environment (Emch et al., 2020; Lang and Smith, 2007; Schwarz et al., 2014).

### 3.5 Antibiotic susceptibility profile of isolates

A total of 107 presumptive *E. coli* and 110 presumptive enterococci colonies were collected from biosolids-amended soil over the course of the study (days 0, 35 and 77) and 14 presumptive *E. coli* colonies from carrots at harvest (day 77). Data from the antibiotic susceptibility testing of the colonies are shown in Table 1. Isolates showed resistance to between zero and all five antibiotics tested. Of the *E. coli* and enterococci colonies collected from antibiotic-supplemented plates, the majority (*n* = 168/179) demonstrated resistance against the respective antibiotics during antibiotic susceptibility testing (Table 1). However, 10.9% (*n* = 10/92) of colonies collected from ampicillin-supplemented plates and 1.1% (*n* = 1/87) of colonies collected from tetracycline-supplemented plates later showed susceptibility to ampicillin and tetracycline disks, respectively. This loss in resistance specifically for ampicillin has been reported previously as well (Palacios et al., 2017). Loss of resistance can occur by a number of factors, particularly if the mechanism comes with a high fitness cost. By growing isolated colonies in a nutrient-rich broth such as Mueller-Hinton without selective pressure, it is plausible that ampicillin- or tetracycline-resistant populations were displaced by susceptible counterparts (Andersson and Hughes, 2011).

**Table 1:** Antibiotic resistance phenotypes of *E. coli* and enterococci colonies isolated from soil amended with biosolids (days 0, 35 and 77) and carrots at harvest (day 77) in a greenhouse study. Note: Soil in pots were amended with biosolids on day 0. On days 0 and 35, soil *E. coli* and enterococci colonies were isolated from agar plates supplemented with antibiotics (ampicillin, tetracycline, or ciprofloxacin for *E. coli* and ampicillin or tetracycline for enterococci). On Day 77, soil and carrot *E. coli* colonies were isolated from non-supplemented plates (no *E. coli* growth was observed on antibiotic-supplemented plates and no enterococci growth was observed on antibiotic-supplemented or non-supplemented plates).

*E. coli* colonies isolated from mTEC agar									
Antibiotic supplement		Ampicillin		Ciprofloxacin		Tetracycline		None	
Day		0	35	0	35	0	35	77 (soil)	77 (carrots)
Resistance phenotype	Ampicillin	19	29	13	1	8	1	6	13
	Chloramphenicol	1	6	13	0	6	0	1	0
	Ciprofloxacin	4	2	29	1	7	0	0	0
	Gentamycin	0	6	5	0	3	0	0	0
	Tetracycline	4	9	22	1	15	0	0	0
	SXT^a^	1	9	14	1	6	0	0	0
Total colonies		20	33	29	1	15	1	8	14
**Enterococci colonies isolated from mEnterococcus agar**									
Antibiotic supplement		Ampicillin		Tetracycline		None			
Day		0	35	0	35	77			
Resistance phenotype	Ampicillin	34		16	18				
	Ciprofloxacin	29		27	36				
	Erythromycin	26		22	31				
	Tetracycline	12		31	40				
	Vancomycin	15		13	21				
Total colonies		39	0	31	40	0			

a Trimethoprim-sulfamethoxazole (SXT).

**Fig. 4:**
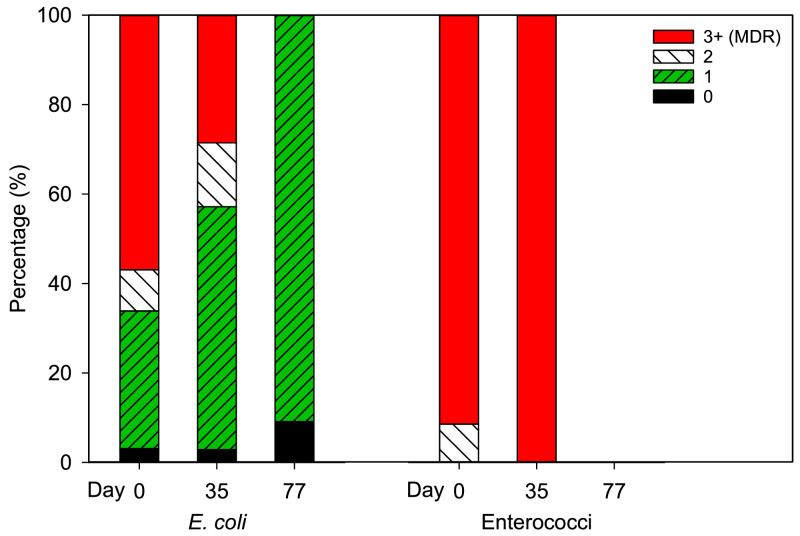
Percentage of *E. coli* and enterococci colonies resistant to 0 (antibiotic susceptible), 1, 2, and 3 or more (multi-drug resistance; MDR) classes of antibiotics. Colonies were isolated from soil (days 0, 35, and 77) and on carrots at harvest (day 77) over 11 weeks in a greenhouse study. Soil in pots were amended with biosolids on day 0. On days 0 and 35, soil *E. coli* and enterococci colonies were isolated from agar plates supplemented with antibiotics (ampicillin, tetracycline, or ciprofloxacin for *E. coli* and ampicillin or tetracycline for enterococci). On day 77, soil and carrot *E. coli* colonies were isolated from non-supplemented plates (no *E. coli* growth was observed on antibiotic-supplemented plates and no enterococci growth was observed on antibiotic-supplemented or non-supplemented plates). A total of 121 *E. coli* (64, 35, and 22 on days 0, 35, and 77, respectively) and 110 enterococci (70, 40, and 0 on days 0, 35, and 77, respectively) colonies were collected from soil. At harvest 14 *E. coli* colonies were collected from carrots.

MDR phenotype was defined as resistance to three or more antibiotics. Percentages of MDR *E. coli* and enterococci colonies as well as those resistant to none (antibiotic-susceptible), one, or two antibiotics during the eleven-week study are shown in Fig. 4. 56.9% (*n* = 37 /65) of *E. coli* colonies collected at the beginning of the study were determined as MDR. Since no culturable *E. coli* were observed in non-amended soils, presumptive *E. coli* isolated from amended soil are most likely from biosolids. On day 35, 22.9% (*n* = 8 /35) of *E. coli* colonies isolated from biosolids-amended soil were MDR. Of the 22 *E. coli* isolated at harvest (in soil and on carrots) none harbored MDR. The decrease in the prevalence of MDR *E. coli* between the three sampling periods from the beginning of the study to harvest was statistically significant (Pearson's chi-squared, *p* < 0.001; Fig. 4). All identified MDR phenotypes are listed in Table S1, with concurrent resistance to ampicillin, trimethoprim-sulfamethoxazole, and tetracycline accounting for 53.3% of MDR *E. coli* isolates. Amongst the 45 total *E. coli* isolates that harbored MDR, 91.1% (*n* = 41) were resistant to tetracycline. Similarly, 80.0 (*n* = 36), 71.1 (*n* = 32), 62.2 (*n* = 28), 44.4 (*n* = 20), and 26.7% (*n* = 12) of MDR *E. coli* isolates were resistant to ciprofloxacin, ampicillin, trimethoprim-sulfamethoxazole, chloramphenicol, and gentamycin, respectively. For enterococci colonies, the prevalence of MDR phenotypes were relatively consistent between day 0 and day 35 (Fisher's exact, *p* > 0.05) and almost all enterococci demonstrated MDR phenotype (*n* = 64 /70 on day 0 and *n* = 40 /40 on day 35). No culturable antibiotic-resistant enterococci were present in soil at harvest. All the 110 enterococci MDR isolates demonstrated resistance to ciprofloxacin, and 98.1 (*n* = 102), 90.4 (*n* = 94), 64.4 (*n* = 67), and 47.1% (*n* = 49) harbored resistance to erythromycin, tetracycline, ampicillin, and vancomycin. The most concurrent MDR phenotypes were resistance to ciprofloxacin, erythromycin, and tetracycline in 88.5% and to ampicillin, ciprofloxacin, erythromycin, and tetracycline in 55.8% of the MDR enterococcus isolates (Table S1).

Overall, our study demonstrates that wastewater effluent irrigation does not impact *E. coli* and enterococci levels in soil and on carrots. Amendment of Class B biosolids, however, significantly increased *E. coli* and enterococci concentrations. In amended soil, almost all *E. coli* were resistant to ampicillin in the beginning of the study and declined quickly to below detection by week seven. Antibiotic-resistant *E. coli* and enterococci decayed at significantly faster rates than total counterparts. *E. coli* were present on carrots at harvest, the majority of which were resistant to ampicillin. Prevalence of MDR *E. coli* declined significantly over time, while MDR enterococci persisted. Since no culturable *E. coli* or enterococci were observed in non-amended soils, antibiotic-resistant phenotypes observed in amended soil are most likely from biosolids. This work is the first to directly compare declining populations of total and antibiotic-resistant *E. coli* and enterococci and the persistence of MDR phenotypes of these enteric bacterial indicators in soils after biosolids amendment and wastewater irrigation. Findings allow for cautious optimism that use of wastewater effluent would not significantly increase antibiotic-resistant enteric bacteria in soil. The survival of fecal indicator bacteria in soil and on harvested carrots, however, indicates the transmission risks associated with biosolids amendment to harvested root crops. The difference in MDR patterns between *E. coli* and enterococci encourages the need for additional studies on the topic. Use of culture-based methods in this study is useful in identifying and quantifying fecal indicator bacteria that actively expressed resistance to the target antibiotics. The limitation of this approach is in the number and types of cultivatable bacteria and target antibiotics. Moreover, the application of culture-based methods does not include antibiotic-resistant bacteria that are viable but non-culturable (i.e., bacteria in VBNC state). Combining culture-dependent and culture-independent methods are needed to give further insight to the fate of enteric ARBs, and their expression of MDR.

## Conclusions

Environmental pollution by antibiotics and ARB is a growing concern as reuse of municipal biosolids and wastewater in agriculture climbs due to shortages of water valuable nutrients. Results of this study revealed that biosolids amendment in soil contributes to significant increases of total and antibiotic-resistant *E. coli* and enterococci at the time of application; however, these levels decrease naturally over time. TWE irrigation did not impact abundance of these bacteria in soil under our study conditions, nor did it affect prevalence of MDR isolates in biosolids amended soil. Total *E. coli* and enterococci persisted in soil for the duration of study (77 days growth season of carrots), and *E. coli* was present on carrots at harvest. Antibiotic-resistant *E. coli* and enterococci decayed faster than total bacterial counterparts and reached the limit of detection before the end of the study. Prevalence of MDR *E. coli* declined significantly over time, while low levels of MDR enterococci persisted. The survival of bacteria in soil and on carrots after biosolids amendment indicates a low level of transmission risk from harvested crops.

## Declaration of Competing Interest

The authors declare that they have no known competing financial interests or personal relationships that could have appeared to influence the work reported in this paper.

## Appendix Supplementary materials

Image, application 1
