# Supplementary material for: Impact of biosolids amendment and wastewater effluent irrigation on enteric antibiotic-resistant bacteria – a greenhouse study
Source: Water Res X. 2021 Sep 8;13:100119. doi: 10.1016/j.wroa.2021.100119 (PMC8452883; doi:10.1016/j.wroa.2021.100119)
Supplement: Supplementary file 1 [file mmc1.docx]

**Supplementary Material**

**Impact of biosolids amendment and wastewater effluent irrigation on enteric antibiotic-resistant bacteria – a greenhouse study**

Catherine Mays^a^, Joy Waite-Cusic^b^, Tyler S. Radniecki^a^, Gabriela L. Garza^a^, Tala Navab-Daneshmand^a,*^

^a^ 105 SW 26^th^ St, 116 Johnson Hall, School of Chemical, Biological, and Environmental Engineering, Oregon State University, Corvallis, OR 97331, United States

^b^ 3051 SW Campus Way, Department of Food Science and Technology, Oregon State University, Corvallis, OR 97331, United States

^*^ Corresponding author: [tala.navab@oregonstate.edu](mailto:tala.navab@oregonstate.edu)

Table S1. Antibiotic resistance phenotypes of multi-drug resistance (MDR) *E. coli* and enterococci colonies isolated from soil amended with biosolids in a greenhouse study.

| Antibiotic resistance phenotype | No. of isolates | % MDR isolates | % Total isolates^a^ |
| --- | --- | --- | --- |
| MDR *E. coli* isolates collected from soil amended with biosolids (days 0 and 35); *n* = 45 | | | |
| Ampicillin, SXT^b^, tetracycline | 24 | 53.3 | 19.8 |
| Ampicillin, ciprofloxacin, tetracycline | 20 | 44.4 | 16.5 |
| Ampicillin, ciprofloxacin, SXT | 19 | 42.2 | 15.7 |
| Ampicillin, ciprofloxacin, SXT, tetracycline | 17 | 37.8 | 14.0 |
| Chloramphenicol, ciprofloxacin, tetracycline | 14 | 31.1 | 11.6 |
| Ampicillin, gentamycin, SXT, tetracycline | 12 | 26.7 | 9.9 |
| Ampicillin, gentamycin, SXT | 12 | 26.7 | 9.9 |
| Ampicillin, gentamycin, tetracycline | 12 | 26.7 | 9.9 |
| Gentamycin, SXT, tetracycline | 12 | 26.7 | 9.9 |
| Ampicillin, ciprofloxacin, gentamycin, SXT, tetracycline | 8 | 17.8 | 6.6 |
| Ampicillin, ciprofloxacin, gentamycin, SXT | 8 | 17.8 | 6.6 |
| Ampicillin, ciprofloxacin, gentamycin, tetracycline | 8 | 17.8 | 6.6 |
| Ampicillin, ciprofloxacin, gentamycin | 8 | 17.8 | 6.6 |
| Ciprofloxacin, gentamycin, SXT, tetracycline | 8 | 17.8 | 6.6 |
| Ciprofloxacin, gentamycin, SXT | 8 | 17.8 | 6.6 |
| Ciprofloxacin, gentamycin, tetracycline | 8 | 17.8 | 6.6 |
| Ampicillin, chloramphenicol, SXT | 6 | 13.3 | 5.0 |
| Ampicillin, chloramphenicol, tetracycline | 6 | 13.3 | 5.0 |
| Ampicillin, chloramphenicol, SXT, tetracycline | 5 | 11.1 | 4.1 |
| Ampicillin, chloramphenicol, ciprofloxacin | 3 | 6.7 | 2.5 |
| Ampicillin, chloramphenicol, gentamycin, SXT | 2 | 4.4 | 1.7 |
| Ampicillin, chloramphenicol, gentamycin, tetracycline | 2 | 4.4 | 1.7 |
| Ampicillin, chloramphenicol, gentamycin | 2 | 4.4 | 1.7 |
| Ampicillin, chloramphenicol, ciprofloxacin, SXT | 2 | 4.4 | 1.7 |
| Ampicillin, chloramphenicol, ciprofloxacin, tetracycline | 2 | 4.4 | 1.7 |
| Chloramphenicol, gentamycin, SXT, tetracycline | 2 | 4.4 | 1.7 |
| Chloramphenicol, ciprofloxacin, SXT | 2 | 4.4 | 1.7 |
| MDR enterococci isolates collected from soil amended with biosolids (days 0 and 35); *n* = 85 | | | |
| Ciprofloxacin, erythromycin, tetracycline | 92 | 88.5 | 83.6 |
| Ampicillin, ciprofloxacin, erythromycin, tetracycline | 58 | 55.8 | 52.7 |
| Ampicillin, ciprofloxacin, tetracycline | 58 | 55.8 | 52.7 |
| Ampicillin, erythromycin, tetracycline | 58 | 55.8 | 52.7 |
| Ampicillin, ciprofloxacin, erythromycin | 47 | 45.2 | 42.7 |
| Ciprofloxacin, erythromycin, vancomycin | 47 | 45.2 | 42.7 |
| Ciprofloxacin, erythromycin, tetracycline, vancomycin | 42 | 40.4 | 38.2 |
| Erythromycin, tetracycline, vancomycin | 42 | 40.4 | 38.2 |
| Ampicillin, ciprofloxacin, erythromycin, vancomycin | 32 | 30.8 | 29.1 |
| Ampicillin, ciprofloxacin, vancomycin | 32 | 30.8 | 29.1 |
| Ampicillin, erythromycin, vancomycin | 32 | 30.8 | 29.1 |
| Ampicillin, ciprofloxacin, erythromycin, tetracycline, vancomycin | 28 | 26.9 | 25.5 |
| Ampicillin, ciprofloxacin, tetracycline, vancomycin | 28 | 26.9 | 25.5 |
| Ampicillin, erythromycin, tetracycline, vancomycin | 28 | 26.9 | 25.5 |
| Ampicillin, tetracycline, vancomycin | 28 | 26.9 | 25.5 |

^a^ Total number of presumptive *E. coli* and enterococci isolates from biosolids amended soil over the course of the study (days 0, 35 and 77) were 121 and 110, respectively, as well as 14 presumptive *E. coli* colonies collected from carrots at harvest (day 77). No MDR phenotypes were observed on day 77.

^b^ Trimethoprim-sulfamethoxazole (SXT)
